# Children’s representations of nature using photovoice and community mapping: perspectives from South Africa

**DOI:** 10.1080/17482631.2017.1333900

**Published:** 2017-07-12

**Authors:** Sabirah Adams, Shazly Savahl, Tobia Fattore

**Affiliations:** ^a^ Department of Psychology, University of the Western Cape, Bellville, Republic of South Africa; ^b^ Department of Sociology, Macquarie University, Sydney, Australia

**Keywords:** Children, visual representations, socio-economic status, photovoice, community mapping, Western Cape, South Africa

## Abstract

The aim of the study was to explore children’s representations and perceptions of natural spaces using photovoice and community mapping. The sample consisted of 28 children aged 12–14 years residing in urban and rural communities in the Western Cape, South Africa. Data were collected by means of a series of six focus groups interviews (three photovoice discussion groups and three community mapping discussion groups). For the photovoice missions, children were provided with a 28-exposure disposable camera and given 1 week to complete their missions. Thematic analysis was employed to analyse the data. Three key themes emerged, namely: safe spaces in nature, unsafe spaces in nature, and children’s favourite places in nature. Socio-economic status (SES) was found to be a determining factor in how children make sense of natural spaces. Children from low SES communities indicated being more constricted in their mobility, and were unable to access to safe natural spaces compared to the children from the middle SES community. It is recommended that an expedient starting point would be to work towards and build environmentally and child-friendly communities for children, with children as key contributors in the planning process using a child participation framework.

## Introduction

The twentieth century has seen a growing body of scholarly research on children’s space and place. With its genesis in the work of philosophers (Aristotle, 1896; Descartes, as, cited in Long, 1969; Ptolemy, as cited in Berggren & Jones, 2000; Plato, 1953) and human geographers (Buttimer, 1976; Relph, 1976, 1981, 1993, Tuan, 1974), understandings of children’s spaces and sense of place have become foregrounded in child research and childhood studies. While various terms are used to describe children’s space and place, researchers and theorists explicate distinct, yet interrelated denotations. It is evident in the literature that although conceptions of place and space vary across academic disciplines, general features of space and place have been established over the years by theorists such as Buttimer (1976), Relph (1976), and Tuan (1977). While space refers more broadly to types of settings for interaction (Philo, 2000; Relph, 1976; Shaw, 1987), place is denoted as a specific site of meaning, which children most often do not convey as ‘children’s place’, but instead they physically reveal these places; a more specific, discernible part of space.

Tuan (1977) similarly accentuated that space is more abstract than place; that what commences in experience as an indistinct space develops into a place as a child experiences a setting, and becomes familiar with it through lived experiences and by assigning particular meanings to it (Tuan, 1977). Low and Altman (1992) note that this affective component and the bond which connects an individual to a particular place embody place attachment. Ensuing theorizations and research on space and place have resulted in a burgeoning field of research (Kyle & Chick, 2007; Rasmussen, 2004) concerned with defining and understanding these terms in general, but more specifically with gaining a greater understanding of children’s sense of space and place.

Another view is put forward by Abbott-Chapman and Robertson (2009), who assert that ‘children’s special places’ are, in fact, cultural constructs which may alter with time, what Haraway (1991, as cited in Instone, 2004) refers to as ‘situated knowledges’. She maintains that these ‘knowledges’ are always historical, located, political, and partial, as the world is at all times articulated from a particular point of view. Children’s favourite places are idealized constructs of places enjoyed and revered—places which aid in regulating negative feelings and coping with perceived stress (Korpela & Ylen, 2007). Commenting from the architectural studies perspective, Najafi and Shariff (2011) indicate that place refers to a strong affective bond between a person and a specific setting. They further maintain that ‘place attachment’, ‘place identity’, and ‘sense of place’ are among concepts that are employed to describe the quality of people’s relationships with a place. Semken and Freeman (2008) theorize further that place attachment and place identity are subsumed within the broader concept of sense of place.

While there has been an increase in research on place attachment over the past 40 years, there is a notable gap in the literature concerning theories of place attachment (Keniger, Gaston, Irvine, & Fuller, 2013; Lachowycz & Jones, 2013; Lewicka, 2011; Zia, Norton, Metcalf, & Hirsch, 2014). The problem inherent in developing a theory of place is the lack of consensus on terminology, as well as a unifying definition of place. A review article by Maria Lewicka (2011) provides a comprehensive account of research on place attachment since its prominence in the work of human geographers over four decades ago. Moreover, the review presents the Tripartite Organizing Framework (Scannell & Gifford, 2010) of place attachment, which emphasizes the significance of ‘person–process–place’ (explicated below). This key review article (Lewicka, 2011) presents a milestone for integrating the otherwise fragmented literature on place research and directions for theory. Lewicka (2011) emphasizes that one of the key gaps in the literature is the process by which individuals develop attachment to places. Lewicka (2011) notes that of 400 published papers on place attachment in over 120 various journals, in excess of 60% were published only in the past decade. In her synthesis of the literature, Lewicka (2011) observes that far more focus has been placed on the person component, with far less work concerning the processes component, which would afford the prospect of understanding the intricacies through which place attachment develops. The following section looks more closely at the Tripartite Organizing Framework by Scannell and Gifford (2010).

### The Tripartite Organizing Framework (Scannell & Gifford, 2010)

The framework considers place attachment as a multidimensional concept encompassing person, psychological (process), and place dimensions. The first dimension is referred to as the actor, the second is the psychological process, and the third is the object of the attachment. These are discussed below.

#### The person dimension (individual and collective place attachment)

While place attachment occurs at the individual and collective (group) levels, with denotations focusing on either of these, Scannell and Gifford (2010) indicate that the two often coincide. The individual level entails the individual connections and links to a place. Research evinces that place attachment is greater when particular locales arouse personal memories (which is believed to further influence a stable sense of self) (Twigger-Ross & Uzzell, 1996); when places are linked to specific memories of experience, such as meeting a significant other or accomplishing a personal milestone. This provides support for individual experiences as a source of place attachment in addition to the physical characteristics of the place (Scannell & Gifford, 2010). At the collective level (group), place attachment includes symbolic meanings of a particular place which is shared among group members. This level of place attachment has been explored across diverse settings and groups, namely cultures, religions, and genders. Scannell and Gifford (2010) thus propose that various meanings at the collective level are influenced by historical circumstances, religion, and other shared experiences among members, with these meanings conveyed to ensuing generations.

#### The psychological process dimension of place attachment

This dimension of place considers the manner in which individuals and groups interact with a place, including the extent of the psychological interactions that take place in the locales or settings that are significant. The literature points to three psychological components which are involved in place attachment or sense of place, that is: affect, cognition, and behaviour.

##### Affect

In terms of affect, several studies show that a predilection for certain places encompasses an emotional affiliation (Scannell & Gifford, 2010). Seminal scholars in the field such as Tuan (1974) proposed the concept of ‘topophilia’, which denotes ‘love of place’, while Relph (1976) referred to the genuine emotional connections individuals have with an environment.

A crucial point is that bonds with places are not inherently positive, as negative experiences in a place can reciprocate negative feelings.

##### Cognition

Cognitive components are also evident in place attachment and includes one’s memories, beliefs, meaning making, and knowledge, which together contribute to the significance of a place. The process of place attachment is also argued to involve one’s sense of self, such as incorporating memories (Scannell & Gifford, 2010). This creation of place meaning has been referred to as ‘symbolic communities’ (Hunter, 1974). The social information in our environments is categorized into schemas (a collection of cognitions), which consist of knowledge and beliefs concerning specific features of oneself (Bartlett, 1932, as cited in Scannell & Gifford, 2010). An individual’s attachment to a place also outlines their distinctiveness, individuality, or similarity in identifying with the place. Similarity is characterized by a ‘sense of belonging’, while distinctiveness refers to unique aspects of an environment (Feldman, 1990; Fullilove, 1996; Stokols & Schumaker, 1981).

##### Behaviour

The final component is that of behaviour, “in which attachment is expressed through actions.” (Scannell & Gifford, 2010, p. 4), and is exemplified in behaviours which conserve an individual’s physical propinquity to a place. This notion of ‘proximity-maintaining behaviours’ is reflected in research which focuses on length of residence. The exploration of the reconstruction of place in post-disaster regions has been another nuance to this line of inquiry. This component of place attachment is thus based on the need to maintain closeness to a place.

#### The place dimension of place attachment

The final dimension of place attachment is the particular place, which has generally been divided into two levels, the social and the physical. Findings show that both the social and physical dimensions of place attachment are significant in the bonding process, inclusive of the spatial level, such as home, neighbourhood, and city. Scannell and Gifford (2010) indicate that emphasis has been placed on the social aspect of place attachment, with the notion that people are attached to places that foster social relations and collective identity. While the physical features of a place are important, when the focus of the attachment is around other people residing in a place and not the physical features of the place it is regarded as a “socially based place bond” (Scannell & Gifford, 2010, p. 4), akin to a ‘sense of community’. Lewicka (2011) notes that attachment to physical dimensions may be stronger than the social dimension in new residents or tourists who visit places, as environmental features develop attachment more rapidly when compared to locals favouring the social dimension. The reasoning behind this is the shorter time it takes to develop and cultivate a bond with natural places than to develop social relationships. Notwithstanding, nature has become the focus of place research in recent years (Scannell & Gifford, 2010). Research has further shown a positive relationship between place identity and environmentally responsible behaviours (Scannell & Gifford, 2010; Vaske & Kobrin, 2001).

### Addressing intersectionality in child research

Given the discussion on space and place, it is crucial to contextualize a particular study. The current study draws together three fields of research, that of child-friendly cities, engagement in nature (environmental psychology), and children’s subjective well-being (SWB), and attempts to amalgamate children’s understandings simultaneously. At this point, it is noteworthy to consider intersectionality, particularly as it relates to child research. Konstantoni, Kustatscher, Emejulu, and Sime (2017, p. 6) argue that childhood studies have been concerned with the manifold “social inequalities and identities in diverse socio-political contexts, but have not considered the politics of intersectionality”.

Initially proposed by Crenshaw (1989), the construct of intersectionality was further developed upon by ‘Black’ feminists in the USA, who critiqued the notion of women as a homogeneous group. The crux of this criticism was that ‘Black’ women’s subjective experiences were influenced by several factors such as race and class in addition to gender. This concept has been applied expansively in gender and feminist studies (Alanen, 2016), and has since been cross-pollinated in various disciplines. Nadan, Spilsbury, and Korbin (2015, p. 43) note further that:

Intersectionality is a theoretical frame-work for understanding how multiple identities such as gender, race and socioeconomic status simultaneously shape human experience at the individual level through interlocking systems of bias and inequality that exist at the macro social-structural level (e.g., sexism, racism, and classism).

Alanen (2016) asserts that while intersectionality has been addressed in research with children, it has not been encompassed in the social studies of childhood, with sparse literature on the topic. However, it is contended that the diversity of children’s subjective experiences has consistently been explored in the social study of childhood to understand the “multitude of children’s childhoods—their lifeworlds, identities, and experiences—but also for analyzing the causal social mechanisms at work.” (Alanen, 2016, p. 160). In a developing context such as South Africa, with one of the highest rates of inequality and child violence in the world, concerns around ethnicity, race, class, gender, and particularly socio-economic status (SES), are crucial. It is thus significant to consider these determinants as they intersect in how children make sense of and understand their lives. In research with children in the context of South Africa, it is crucial to remain cognizant of the intersection of key issues such as gender, SES, and inequality, which reflects the heterogeneous nature of childhood and evinces nuances in their experiences (Alanen, 2016).

Kjørholt (2003, p. 265) contends that “Children’s special places have been connected to place identity and attachment to place, to creativity, to the need for children to find a place of peace and ‘refuge’ from the adult world, to closeness to nature, and as places for ecstatic experiences and more”. Chawla (2000) speaks of ‘places of conviviality’, which refer to busy public or commercial places; ‘places of solidarity’, which demonstrate that others acknowledge one’s existence and confirm one’s rights and needs; and finally, ‘places of possibility’, which indicates that children’s special places do not only exist in the present; they may also exist in the imagination as prospects for the future (Chawla, 2000). A number of studies show that children often identify natural spaces as their special or favourite places (Adams & Savahl, 2015; Chawla, 2006; Sancar & Severcan, 2010).

### The concept of nature

Malone (2016) notes that the ‘new nature movement’, the ‘children in nature’ movement and the ’nature–outdoor education movement’ have recently re-emerged in the public sphere. These movements have predominantly been founded on the contention that children are not afforded sufficient opportunities to engage in nature, based on growing fear of social hazards and ‘stranger danger’. While it is argued that within the contemporary Western cultural vernacular the conflation of children with an “idealized form of pure nature” (Taylor, 2011, p. 423) has become a prevailing trend in current thinking and research (Taylor, 2011), Malone (2016) maintains that this idealism should be challenged. She notes that the culmination of three points, namely viewing humans as inherently close to nature, considering modern life as disconnected from nature, and the lack of engagement in nature provide credence to a particular profile of the human/nature relationship. This relationship could potentially be characterized as humans being to some degree ‘more or less’ nature, as ‘connected or disconnected from nature’, and able to ‘dominate nature’. Malone (2016, p. 43) thus argues that:

Rather than continuing to reinforce these views, I am provoked to contemplate the possibilities that exist to challenge these enduring perceptions that position humans as exceptional. What new theoretical approaches, for instance, would be useful to deconstruct human/nature, object/subject binaries and promote more inclusive means of describing the nature–human collective, and to move away from cultural universalisms about the natured child?

It is thus critical to note that ‘nature’ is not a unitary concept; Adams and Savahl (2016b, p. 11) thus assert that there are “gradations in children’s conceptualisation of the construct of nature.” Linzmayer and Halpenny (2013) elaborate on this, and advance the definition of nature as comprising anthropogenic amendments on the one hand, and on the other demonstrating social and cultural influences, maintaining the social construction of the concept of nature.

The opacity as to whether nature includes humans is a long-standing debate with an historical focus, indicative that established social and cultural politics are entrenched in these delineations (Macnaghten, 1993). Macnaghten (1993) avers the contestation around three coterminous denotations: firstly, that of intrinsic nature, referring to the elementary characteristics of a ‘thing’ (e.g. the nature of childhood); secondly, that of external nature, referring to ‘nature’ as the pristine or ‘untouched’ material world external to humanity (e.g. the natural environment); and finally, universal nature making reference to universal law or reality, which may or may not include humans (e.g. ‘natural’ laws or ‘Mother nature’) (Macnaghten, 1993). Essentially, then, the debate around the operationalization of nature is along two divergent trends: the first is the criticism of the ‘all-inclusive’, absolute view of nature, and the second argues for centrality of the concept (Attfield, 2006). Some scholars (e.g. Giddens, 1994; Attfield, 2006; McKibben, 1990; Merchant, 1990 as cited in Attfield, 2006)) claim that nature has become socialized, and extend this argument to assert that “nature no longer exists”—that we have reached what McKibben (1990) refers to as “the end of nature” (p. 11). McKibben’s (1990) argument resonates with the contemporary criticism that nature, in terms of entities untouched by humanity, no longer exists. Crist (2004) critiques the postmodern constructionist view of nature, contending that the social construction of nature is “narrow and politically unpalatable” (p. 6). She maintains that while constructionists endeavour to unearth the socio-cultural genesis, they do not deconstruct their own rhetoric.

The child’s freedom to construct places of their own presupposes a safe centre from which to depart (Chawla, 2000; Sancar & Severcan, 2010). Sancar and Severcan (2010) elaborate further that a well-developed and fostered sense of place is critical for children’s well-being. Tuan (1977) hypothesized that when children observe their treasured places being degraded or polluted, this can damage their lifeworlds. The probable negative outcomes of this tainting of a child’s special place may result in dissonance, loneliness, a heightened sense of fear, unhappiness, and behavioural disorders (Brown & Perkins, 1992, as cited in Sancar & Severcan, 2010). Hay (1998) further argues that a sense of place is not developed in children whose mobility is constrained. A prominent factor that has limited children’s play in natural space and place is the pervading number of social hazards in their neighbourhoods and communities, in both developed and developing countries (MacDougall, Schiller, & Darbyshire, 2009; Malone & Hasluck, 2002; Swart-Kruger & Chawla, 2002; Wals, 1994). Due to these hazards which are present in children’s lives, they are not able to exercise their right to play safely within nature. The social hazards most frequently mentioned by children are traffic, ‘stranger danger’, limits to mobility and accessibility, and issues around safety.

The case of South Africa, with its unique history of apartheid, has resulted in a society which is characterized by high levels of crime and violence, presenting particular threats to children’s safety. Safe natural spaces for children to build memories and care for special places is limited and especially challenging in impoverished communities (see Adams & Savahl, 2015; Parkes, 2007). Previous participatory studies with children conducted in South Africa have identified safety as a paramount concern in children’s lives (Adams & Savahl, 2015; Isaacs & Savahl, 2014; Parkes, 2007; Savahl, Malcolm, et al., 2015). Furthermore, these local studies (Adams & Savahl, 2015, 2016b; Parkes, 2007; Savahl, Malcolm, et al., 2015) show that negative outcomes are exacerbated when children experience violence and threats to safety first-hand in natural spaces. Credible threats to children’s safety are evident in the high rates of reported incidents of abuse, sexual violence, kidnapping, and murder enacted against children in the country (South African Child Gauge, 2014). It is therefore crucial to consult and include children in research which seeks to understand and explore their subjective perceptions of the spaces that are allocated to or meant for children in their communities, to ascertain what these spaces mean to them, and how they use them.

### Visual methodologies in research with children

The focus on participatory methodologies with children to explore their environments has gained substantial momentum in recent years (see Amsden & VanWynsberghe, 2005). This focus has been greatly influenced by the global drive among states parties which have ratified the United Nations Convention on the Rights of the Child (UNCRC) to advocate for, and employ participatory techniques that empower children. Among an array of participatory techniques, photovoice and community mapping have been used extensively in empirical studies with children, emphasizing children’s subjective perceptions and evaluations of their lives and neighbourhoods (e.g. MacDougall et al., 2009; Sancar & Severcan, 2010).

Photovoice, previously termed photo novella (Wang & Burris, 1994, 1997), has, in particular, been used to explore children’s engagement with natural places (e.g. Castonguay & Jutras, 2009; Rasmussen, 2004). A systematic review on research focusing on children’s environmental views by Adams and Savahl (2016a) found that the most commonly used data collection techniques across qualitative studies were drawings and mapping techniques (primarily with children between the ages of 5 and 7 years) (Kopnina, 2011; Malone & Tranter, 2003); observations (with children aged 7–18 years) (Malone & Tranter, 2003; Palmberg & Kuru, 2000); and interviews, both in-depth and focus groups (with children between the ages of 5 and 12 years) (Adams & Savahl, 2015; Hordyk, Dulude, & Shema, 2014; Kong, 2000). Among the photo-elicitation techniques, the participatory technique of photovoice in particular was used in the majority of these studies (Burke, 2005; Rasmussen, 2004), affording children the status of active participants within the research process. Through this participatory research method, children are considered as collaborators who possess agency, and are given a platform to present photo journeys of neighbourhood experiences. It is evident from the literature that photovoice as a participatory method has been employed increasingly with children across diverse contexts (e.g. Kopnina, 2011; Malone & Tranter, 2003; Sancar & Severcan, 2010; Strack, Magill, & McDonagh, 2004; Zuch, Mathews, De Koker, Mtshizana, & Mason-Jones, 2013). In a recent edition of the *International Journal of Qualitative Studies on Health and Well-being*, a study by Benninger and Savahl (2016) sought to explore children’s constructions of the self across two urban communities in Cape Town, South Africa, using photovoice and community mapping. Similarly, a study by Malone (2016) explored children’s encounters with place in La Paz, Bolivia, using a posthumanist approach using the photovoice method.

The significance of photovoice is captured in the conjecture by Wang and Redwood-Jones (2001, p. 560) that, “Photovoice is a powerful photographic technique that enables people to assess the strengths and concerns of their community and communicate their views to policymakers”. Delgado (2015) notes that it is significant to emphasize the distinctions between photo elicitation and photovoice, as the former is a research method which may form part of “a wide variety of methodologies” (p. 7), while photovoice draws on participatory principles and is housed in methodology akin to community-based action research. With photographs being intricate, and interpretable in a number of ways (Radley, 2010), the use of photovoice in this study provides the opportunity to explore the potential meanings behind children’s representations from children directly.

### Aim of the study

The aim of the study was thus to explore children’s representations and perceptions of natural spaces using photovoice and community mapping.

## Method

### Research design

The current study forms part of and reports on data from the multinational qualitative project Children’s Understandings of Well-Being (CUWB): Global and Local Contexts (Adams & Savahl, 2016b; Fattore, Fegter, & Hunner-Kreisel, 2016). The aim of the CUWB project is to explore children’s subjective understandings of well-being and different aspects of their lives across 21 countries using participatory research methods. Data were collected by means of three interrelated sequential data collection techniques, namely focus group interviews, photovoice, and community mapping. The current study, however, reports only on the results from the photovoice and community mapping with children, which employed a qualitative methodological framework to understand children’s representations and how they make sense of natural spaces using photovoice and community mapping. Findings from the focus groups using discourse analysis can be found in Adams and Savahl (2016b).

### Research context

**Figure 1.**
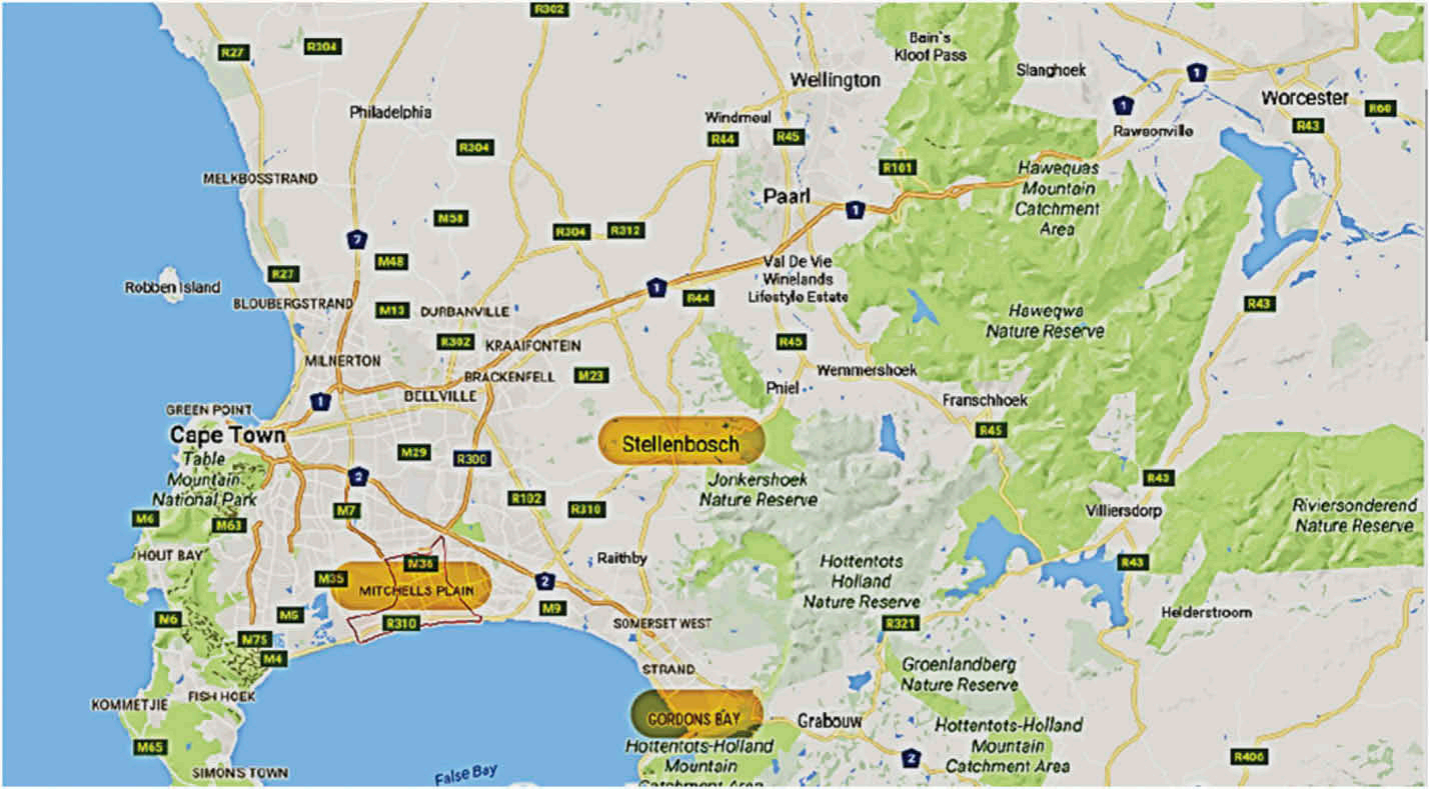
Study research contexts mapped

The study was conducted in three areas diverse contexts in the Western Cape Province of South Africa; namely Gordon’s Bay, Mitchell’s Plain, and Stellenbosch. Gordon’s Bay is a town located in the Overberg region of the Western Cape. Mitchell’s Plain is a suburb within the City of Cape Town located in the Western Cape with a number of sub-districts, while Stellenbosch is a local municipality comprising smaller suburbs and located in the Cape Winelands region of the Western Cape. These three areas are further explicated below. Figure 1 below presents the location and proximity of these three areas.

#### Gordon’s Bay

Gordon’s Bay is a coastal town located approximately 54 km from the Cape Town city centre. The population was estimated to be 15 786, with predominantly ‘White’ residents. Key indicators show that majority of the population live in formal housing with access to basic services, and have completed secondary schooling or higher, with most households falling within the R12 801–R25 600 income bracket. The crime rate for 2013–2014 was substantially lower than national estimates, with the majority of reported crimes consisting of common assault, burglary, and a low reported incidence of sexual crimes and murders (South African Police Services, 2014).

#### Mitchell’s Plain

Mitchell’s Plain is situated approximately 32 km from the Cape Town city centre, and has been identified as one of the most dangerous areas in South Africa with the highest incidence of reported crimes (www.crimestatssa.com). The population was estimated to be at 310 485, and the majority classified as ‘Coloured’ (Statistics South Africa, 2011). National estimates show that only just over one-third of the population have completed secondary education or higher. Thirty-eight per cent of households have a monthly income of R3200 or less, with the majority living in formal housing. Although national census data shows that the vast majority have access to basic services, the suburb is characterized by a range of socio-economic problems.

#### Stellenbosch

The Stellenbosch Municipality is situated in the centre of the Cape Winelands, approximately 50 km from the Cape Town city centre. The municipality has an estimated population of 155 753, with the majority classified as ‘Coloured’. Forty-three per cent have completed secondary education or higher, while 3.1% have not completed any formal schooling. The majority of the population live in formal housing and have access to basic  amenities (Statistics South Africa, 2011). Nationally, Stellenbosch is ranked among the top 10 areas with the highest incidence of reported crimes, evincing among the highest incidence of burglary, theft from motor vehicles, commercial crime, and robbery (www.crimestatssa.com).

### Participants and sampling

The total sample consisted of 28 children between the ages of 12 and 14 years, purposively selected from three primary schools in low- and middle-income communities, situated in rural and urban geographical locations in the Western Cape of South Africa. Three groups of children were selected from the three schools: two of the groups consisted of 10 participants each (one group from Seaview, comprising five girls and five boys; and one group from Gordon’s Bay, nine girls and one boy), with the third group consisting of eight participants (Stellenbosch: five girls and three boys). While it was envisaged to obtain an equal gender sample of girls and boys from each school, owing to the voluntary nature of participation this was not always possible. The motivation for selecting this age cohort was due to the identification in the literature that children of this age group are more likely to assess their own behaviour and the impact of their subsequent actions upon the environment (Wilson, 1996). The primary motivation for the final selection of the three participating schools was dependent on whether they offered access to children from different racial, cultural, language, and socio-economic backgrounds. Additional inclusion criteria for participants included perceived reliability, enthusiasm, and willingness to participate in the study. The staff liaison at each school assisted with the recruitment of learners, using the aforementioned inclusion criteria.

### Data collection

Data were collected using two participatory techniques, namely photovoice and community mapping. A total of six discussion groups were held with the participants.

### Photovoice

“Photovoice enables people to identify, represent, and enhance their community through a specific photographic technique” (Wang, 1999, p. 185). In the current study one photovoice training session was held, and one photovoice feedback session where participants discussed their printed photographs with the research team. Children were asked to take photographs of the places that make them happy and unhappy, as well as their favourite places in nature. They were each provided with a 24-exposure disposable camera and given a period of 7 days to conduct their photovoice missions. Before carrying out their photovoice missions, group discussions were held with the participants that focused on the natural spaces in which they engage. Potential research questions were then collaboratively formulated with the participants. The participants were also trained in basic camera and photography techniques, and familiarized with the ethical employment of community photography, such as the use of cameras, power, and the responsibility and authority conferred on participants with cameras (Wang & Redwood-Jones, 2001). The following photovoice ethics guidelines were followed to ensure the participants’ and community members’ privacy (Wang & Redwood-Jones, 2001). The participants were made aware of respect for privacy law against four distinct types of invasion: intrusion into one’s private space, disclosure of embarrassing facts about individuals, being placed in a false light by images, and protection against the use of a person’s likeness for commercial benefit (Wang & Redwood-Jones, 2001). To address intrusion into one's private space two written consent forms were administered to the participants. The first addressed the general ethics protocols of the authors’ university institutional review boards and detailed the particulars of the study. The second required the participants to obtain permission, by way of a signature, from the individual being photographed prior to taking any photographs. A third consent form was administered once the photographs were printed, in which the participants gave permission for their photographs to be published and used in the study. Wang and Redwood-Jones (2001) further discuss that the safety of the participants must be a fundamental consideration.

The photovoice mission session was carried out by the children independently after school, for which they were accompanied by either an older sibling or a parent. As proposed by Wang and Burris (1994), the group discussions were facilitated by the following questions: What do you see here? What is really happening here? How does this relate to our lives? Why does this problem or strength exist? and What can we do about it? An ensuing group discussion was conducted whereby the participants provided narratives expounding on the significance and the meanings their pictures hold for them, which was followed by group views of what the photograph represents.

### Community mapping

Community mapping was utilised as a visual data collection technique which provided unique representations of children’s worlds in this study. Amsden and VanWynsberghe (2005) note that community mapping may be used to document geographically significant spaces and places, as well as additional varieties of abstract data. The abstract data and intricacies in children’s maps are made sense of when children provide in-depth narratives for the detail therein. Widely considered as an empowerment and child-centred technique, community mapping is foregrounded on “validating the knowledge and experiences of participants” (Amsden & VanWynsberghe, 2005, p. 361). Additionally, given the participatory nature of this technique, it is considered to address the issue of power dynamics and inequities present in the research–participant relationship. This data collection technique was complemented by the photovoice and focus group interviews (reported elsewhere).

### Data analysis

The discussion groups with the children about their photographs and maps were analysed using thematic analysis. More speciﬁcally, Braun and Clarke’s (2006) six-phase guide to undertaking a theoretical thematic analysis was employed. Theoretical thematic analysis is closely related to the researcher’s theoretical proclivities and is usually coded to align with the study’s research aims. Phase one, familiarizing oneself with the data, involved an immersion in the data which was characterized by repeated readings of the transcripts. In phase two, the initial codes were generated, followed by phase three, which focused on the identiﬁcation of the themes based on the initial codes. In phase four, the themes were reviewed and reﬁned, with phase five entailing deﬁning and the ﬁnal naming of the themes. Phase six focused on the production of the study findings based on the ﬁrst ﬁve phases of analysis.

### Procedure and ethics

Ethics clearance to conduct the study was obtained from the Senate Research and Ethics Committee at the University of the Western Cape. Once permission was gained from the principals of the respective schools, ethics clearance was sought from the Western Cape Education Department (WCED). Children who were interested in participating were recruited by the grade 6 head of department of each school, and at one school, by the school counsellor. Children were only able to participate if signed consent was obtained from their parent or guardian, and from the children themselves. An initial session was held with the participants to inform them of the purpose and aim of the study, what their participation would entail, as well as the core ethics principles of informed consent, voluntary participation, confidentiality, and the right to withdraw from the study at any time without negative consequences. The participants were requested to keep the content and discussions that took place within the discussion sessions confidential. The sessions were audio-recorded, with the participant’s permission, and transcribed verbatim. The transcribed texts were verified by a research psychologist external to the study. The participants were also informed that the data gathered would be used for a monograph thesis which would be publicly available, as well as peer-reviewed publications and conference presentations. Focus group discussions were conducted on the school premises during administration sessions at the beginning of the school day and after school. They were conducted by the primary researchers and assisted by a co-facilitator.

## Findings

The study aimed to explore children’s representations and perceptions of natural spaces, using photovoice and community mapping. These two participatory techniques were employed to capture participants’ reflections on significant spaces and places, and photo journeys and mapping to explore neighbourhood experiences and perceptions of natural spaces. The study was conducted in three socio-economically diverse communities in the Western Cape of South Africa, with children’s understandings and experiences evincing a diversity of ‘childhood’. Three key themes emerged from children’s discussions about their photographs and mapping using thematic analysis, namely safe spaces in nature, unsafe spaces in nature, and children's favourite places in nature. SES was found to be a determining factor in children’s identification of safe and unsafe spaces in their communities, as well as their favourite places in nature. These themes are discussed in detail below.

### Safe spaces in nature

**Figure 2.**
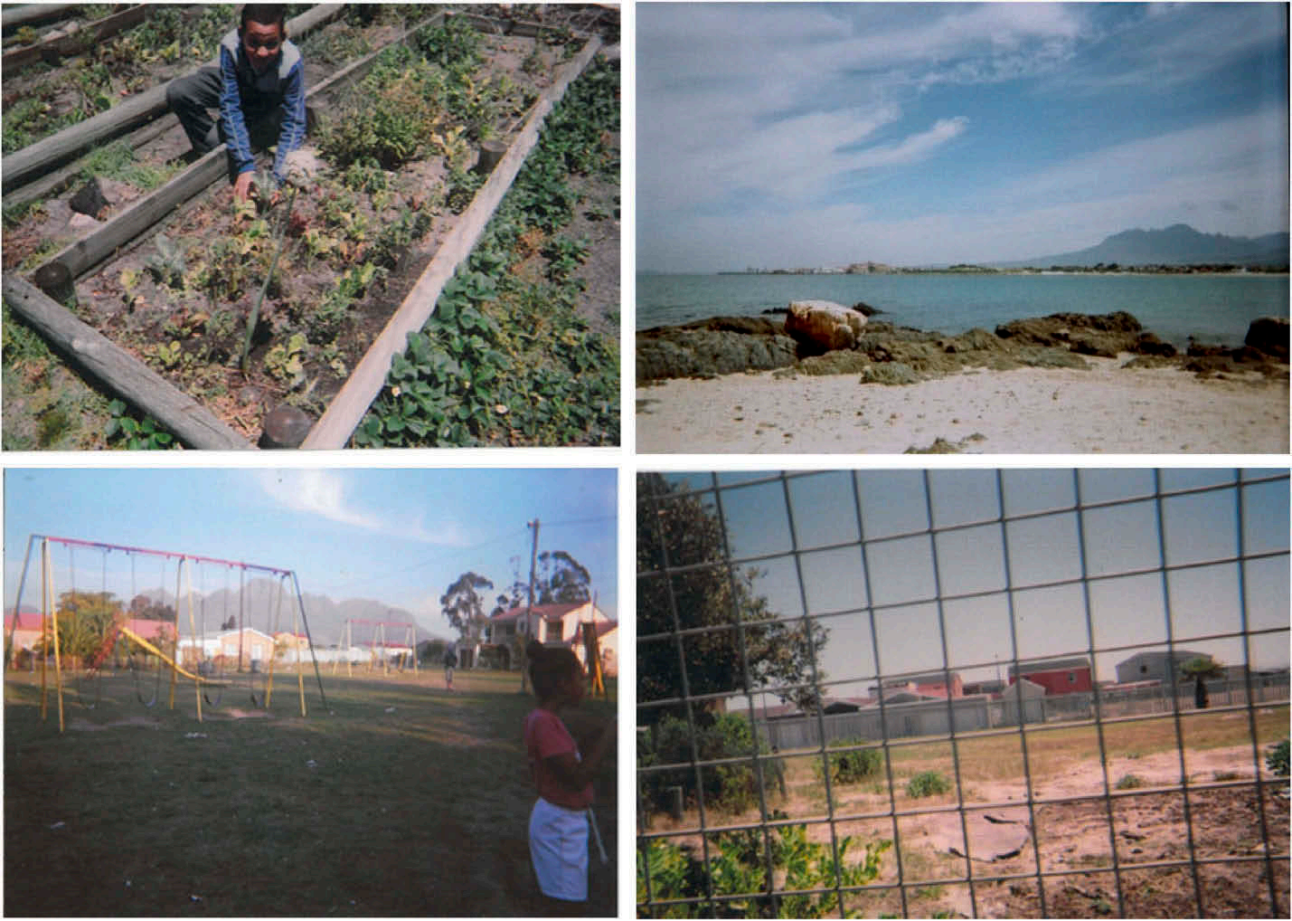
Pictures depicting the safe natural spaces in children’s communities, such as school gardens (top left, Mitchell’s Plain), the nearby beach (top right, Gordon’s Bay), the park (bottom left, Mitchell’s Plain), and their school grounds (bottom right, Stellenbosch).

**Figure 3.**
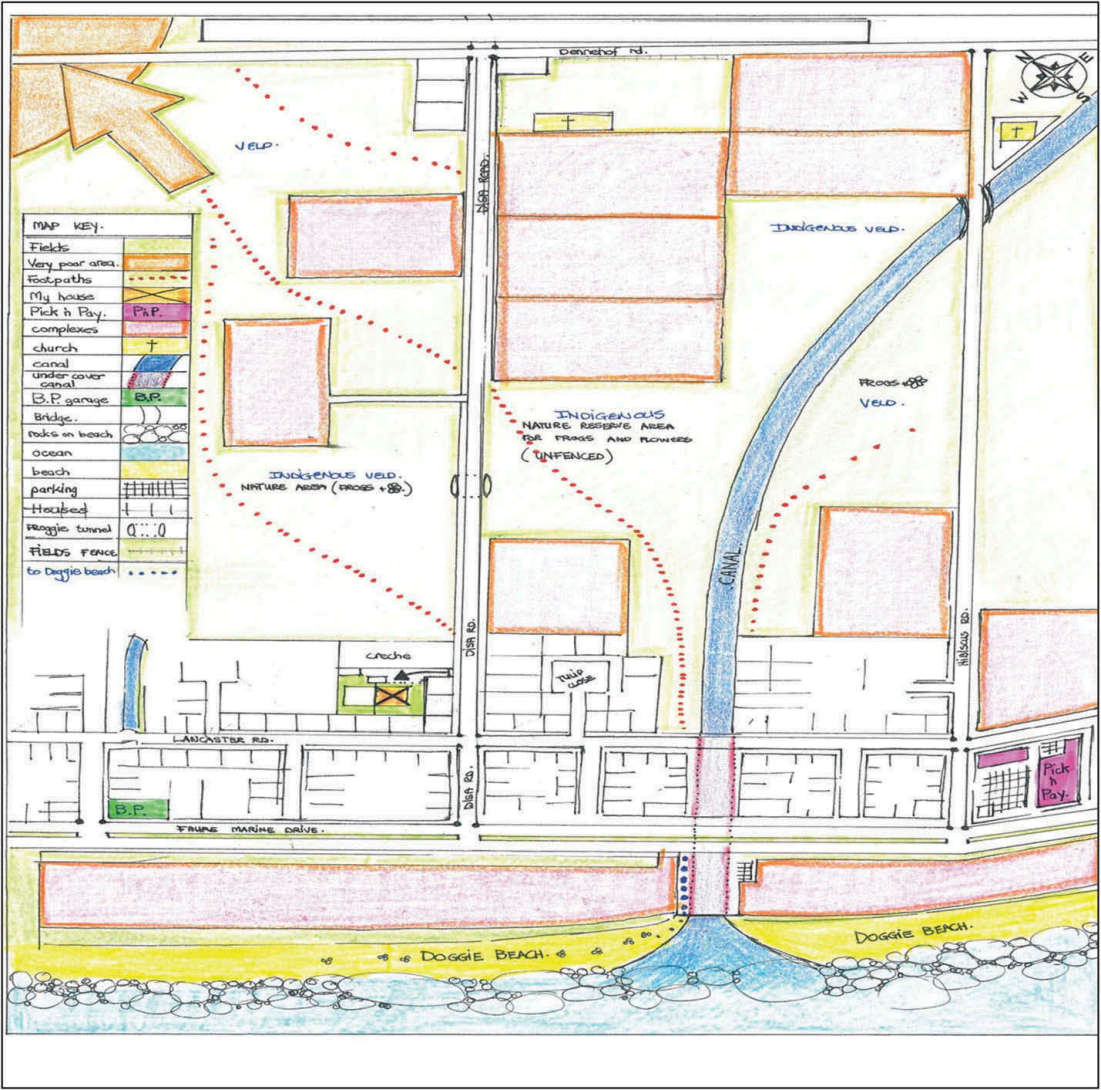
This intricate map was sketched by a male participant from Gordon’s Bay, showing his excellent attention to detail and knowledge of his neighbourhood, particularly natural places.

There were great disparities in children’s perceptions and experiences of safe spaces to engage in within the three communities of Mitchell’s Plain, Stellenbosch, and Gordon’s Bay. The socio-economic standing of the community in which children resided played a key role in how they made sense of, assigned meaning to, and experienced natural spaces, which was expressed through their use of photovoice and community mapping and subsequent discussion groups (Figures 2 and 3). For the mapping exercise children used two stickers: red stickers represented unhappy and unsafe spaces, and gold stars showed safe and favourite places. For Gordon’s Bay, none of the maps had red stickers indicating unsafe spaces, while children’s maps from Mitchell’s Plain and Stellenbosch were populated with red stickers indicative of unhappy and unsafe spaces. Children’s appraisal of their community showed that they had mixed emotions about the different environments in their community, where safe spaces were synonymous with positive emotions, and unsafe spaces synonymous with negative emotions. Children revealed that the safest spaces for them which enabled safe play in natural spaces were at home (in gardens and backyards), at school, on the soccer field, and at the beach (this was mentioned only by the children from Gordon’s Bay, and was only possible if accompanied by an adult). The library, and places of worship were also mentioned as safe spaces. For many children, the safe places they referred to, for example, particular parks or fields, were not safe all of the time—these places were seen to be unsafe at certain times of the day or week. One of the participants articulated that the soccer field photographed in his area is especially safe, stating that “the adults watch over us”. Further discussion about this photograph indicated that children’s safety in this space was contingent on adult supervision. However, there were instances when these spaces were compromised.

The concern for safety was more prominent in children's understandings from the low SES communities. Very often children’s photographs and maps from the low SES (Mitchell’s Plain) and rural community (Stellenbosch) showed the safest natural places to be close to home, such as their own backyard, an open field across from their home, or their school playground. Notwithstanding the perilous neighbourhoods in which most of the children live, their intricate knowledge of their communities enabled them to navigate their way safely through their communities. This, however, was not achieved without a sense of trepidation and anxiety. Most of the children from Gordon’s Bay, in contrast, were in a position to negotiate their mobility, as evinced in their photographs and maps, with many of them able to explore their environments and nature independently. These children’s photographs and maps showed the diverse natural spaces that they are able to navigate, with their favourite places in nature further from home, away from adult supervision. This is best demonstrated in Extract 1 below.

#### Extract 1

**Female participant:** I also like cycling in the mountains.

**Female participant:** Then you like feel away from everything you can just be like yourself … Get away from all the electronic stuff and worries …

**Female participant:** Where we camped last time it was like a river and then you walk across the river and it’s the sea so you could go to the river or the sea.

(**Group 1: Session 1; Gordon’s Bay)**

In comparison, the children from Stellenbosch and Mitchell’s Plain were more restricted, especially girls. Aside from school being a safe space for many children, most made mention of only one other safe space for them in their community, with most of their discussions centring upon first-hand experiences of abuse or violence. This is best demonstrated in Extracts 1 and 2 from the different SES communities.

There was consensus among all the children that school was a safe space, offering safe natural spaces for children. The sense of attachment to school varied. Children from the rural school in Stellenbosch indicated that they were especially happy at school, as their home environments posed numerous threats against them. For many of them, these threats were within their own home. While the children from Mitchell’s Plain also considered school a safe space, they were unhappy that they were no longer allowed to play on the open field or in the garden at their school, which had recently been gated off from students. While many of their pictures were taken at the vegetable garden at school, this was done after school when teachers would not deny them entry. It was for this reason, coupled with children’s limited mobility in their unsafe communities, which led them to assert that “school is like a prison”, as captured in the photograph in Figure 2 (bottom right). While school was considered a safe space for children, the commute to school was not always safe. Some children discussed having to cross a park to get to school, with a male participant commenting that “you just don’t know when they gonna start shooting”; again highlighting the importance of safety in children’s lives. It further accentuated the impact that this dangerous space in their community has on their daily living and their SWB.

Many children across the three communities spoke about the garden at home being a safe natural space where they enjoyed spending time. A male participant expressed his predilection for this activity in mentioning that he “talks to the plant” to foster its growth. Other children spoke about how they also spend leisure time in their backyards among the trees and plants, which made them feel “calm” while doing their homework in nature. Conversely, one of the main reasons children gave for staying indoors was the workload at school, and having to complete homework. A female participant’s discontent with being indoors at home was evident in her statement that “When I’m inside I dunno why but I’m moody”. In comparison to many of the male participants who preferred staying indoors and playing console or computer games, the female participants preferred being outdoors but did not often have the opportunity to do so. An interesting finding from the photovoice session was the photographs taken by one participant in a plant nursery as he had limited safe, green space to utilize and engage in. In addition, while children from Gordon’s Bay had an abundance of safe natural spaces at home and in their community, a female participant took photographs of a lifestyle living complex which boasted a dam. While these safe natural spaces formed the basis of children’s play spaces, the lack of mobility and ability to explore other natural spaces in their neighbourhood influenced children in profound ways. In relation to her dissatisfaction with the experience of being home-bound owing to the unsafe neighbourhood, a female participant articulated that “At home you feel crowded by houses”, expressing the need to be in nature; to be free and independent. A different perspective provided by a female participant from Gordon’s Bay was that, “It’s always so nice to be outside in the fresh air”, thereby expressing her frequent engagement and direct experiences in nature reflected in the word “always”. It is discernible from children’s understandings that the natural places in which they engaged and spent time have an effect on their quality of life, which in turn reflects the quality of their environments.

### Unsafe spaces in nature

There were several unsafe natural spaces which children discussed in the photovoice and mapping sessions. In the mapping exercise for one of the schools, children did not depict any favourite natural spaces, and instead predominantly portrayed unsafe spaces in their community. As Myers (2014) critically notes, the levels of crime and violence in children’s communities are significant identifiers of children’s level of mobility and their perceptions of safety. In the three communities in this study, children made sense of unsafe spaces in variant ways, again with children from the low SES communities citing numerous unsafe spaces in their neighbourhoods. Most children’s immediate environments were unsafe. Consultations with children about their maps showed how they strategically traversed and plotted their way through their neighbourhood for both school and leisure activities. Children showed that almost all natural spaces in their communities are unsafe, such as open fields of green space, parks, rivers, beaches, picnic areas, and ‘wild’ nature. One of the examples of ‘wild’ nature was the presence of baboons in the mountainous areas close to where the children from Gordon’s Bay live. According to these children, these animals can become violent if they feel threatened or are provoked, with one participant mentioning that in his encounter the baboons threw stones at him.

**Figure 4.**
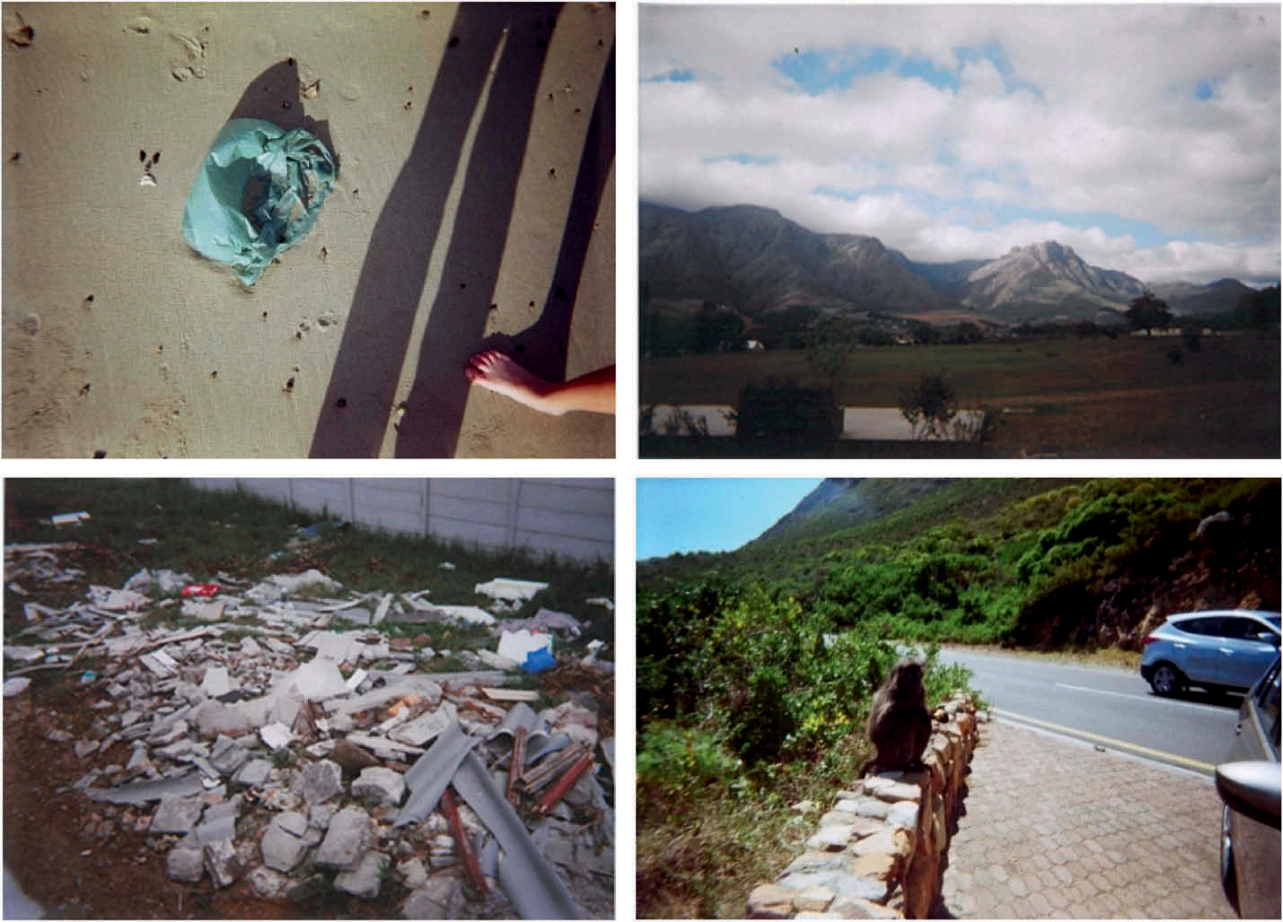
Photographs showing the unsafe spaces in children’s communities. In particular, we see a littered field (where the potential for children being assaulted or kidnapped is high), open green space which children are unable to access, and ‘wild’ nature (evident in the picture with the baboon).

Children’s understandings of these unsafe spaces was intricately linked to their characteristically unsafe neighbourhoods. The communities of Mitchell’s Plain and Stellenbosch have among the top 10 highest crime rates in the Western Cape, which was clearly demonstrated in children’s discussions of these communities. The difference in SES of the children’s communities resulted in stark variances in children’s photographs and maps, evident in Figures 2 and 4. Ultimately, what emerged from children's perspectives from the low SES communities was that there are no safe natural spaces for children in their communities. While concerns for children’s safety is prevalent in all communities, children in more affluent, ‘safer’ areas had more opportunities to explore their natural surroundings.

#### Extract 2

**Facilitator:** So … why are you indoors a lot?

**Male participant:** It is safer inside than to be outside.

**Female participant:** Because of the violence.

**Male participant:** They shoot a lot.

**Male participant:** The people are gang- related there.

**Male participant:** It is actually ourselves that is worried about it.

(**Group 1:** **Session 1; Mitchell’s Plain)**

**Female participant**: My dad says I’m not allowed to go in because when we first moved here it was still safe and now my dad says we not allowed if we at home the doors have to be closed and all the windows have to be locked because there are a lot of robberies happening out there.

**Female participant:** My mom is very protective of me walking around although lately she has lightened up and like … the other day we walked home for the first time ever.

(**Group 1: Session 1; Gordon’s Bay)**

**Figure 5.**
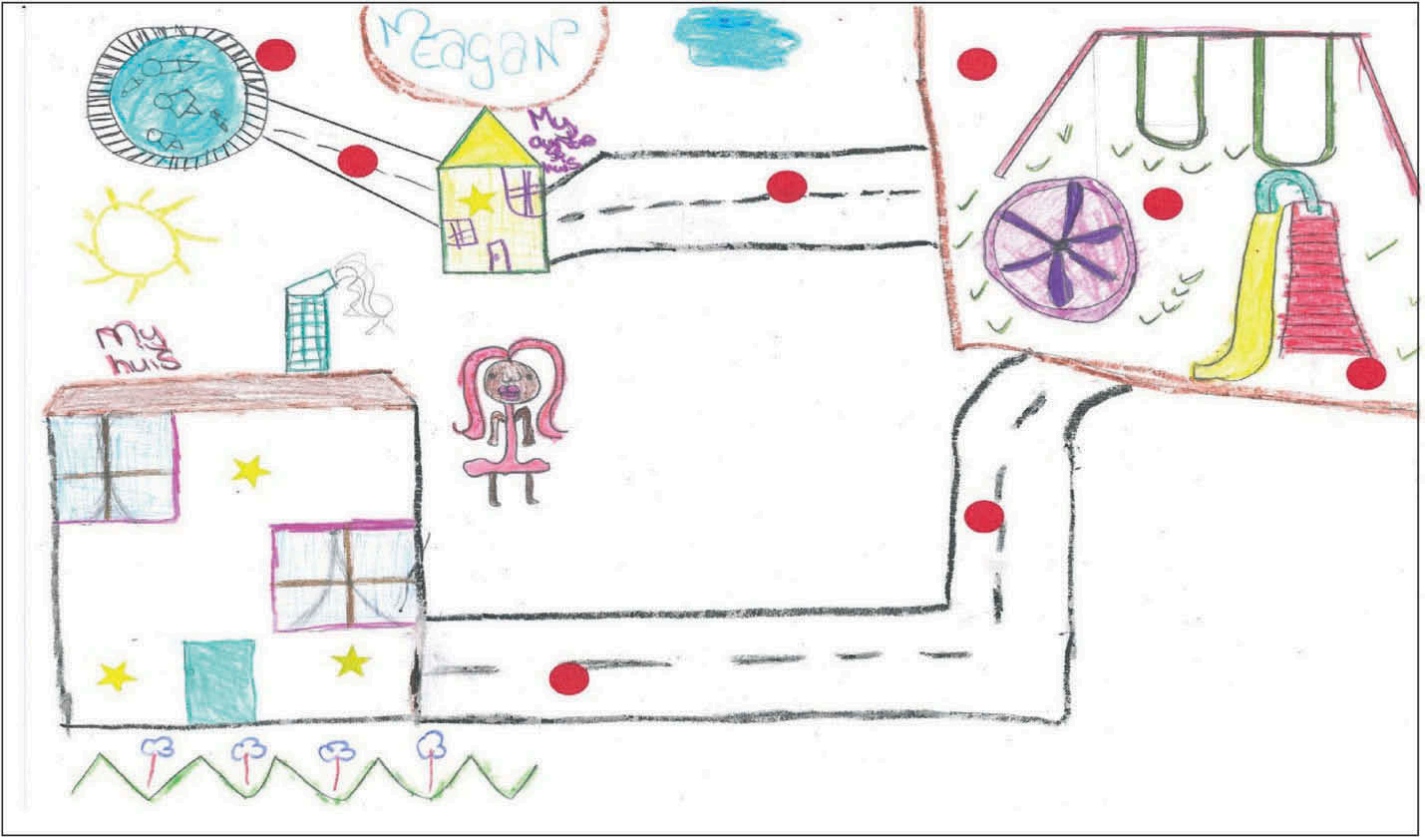
In Map 3, we can see that there are only two safe spaces for this participant: her house and her friend’s house (demonstrated by the gold stars). It is also evident that the parks, the roads, and the beach, which are all spaces surrounding and close to home, are all unsafe (indicated by the red dots).

**Figure 6.**
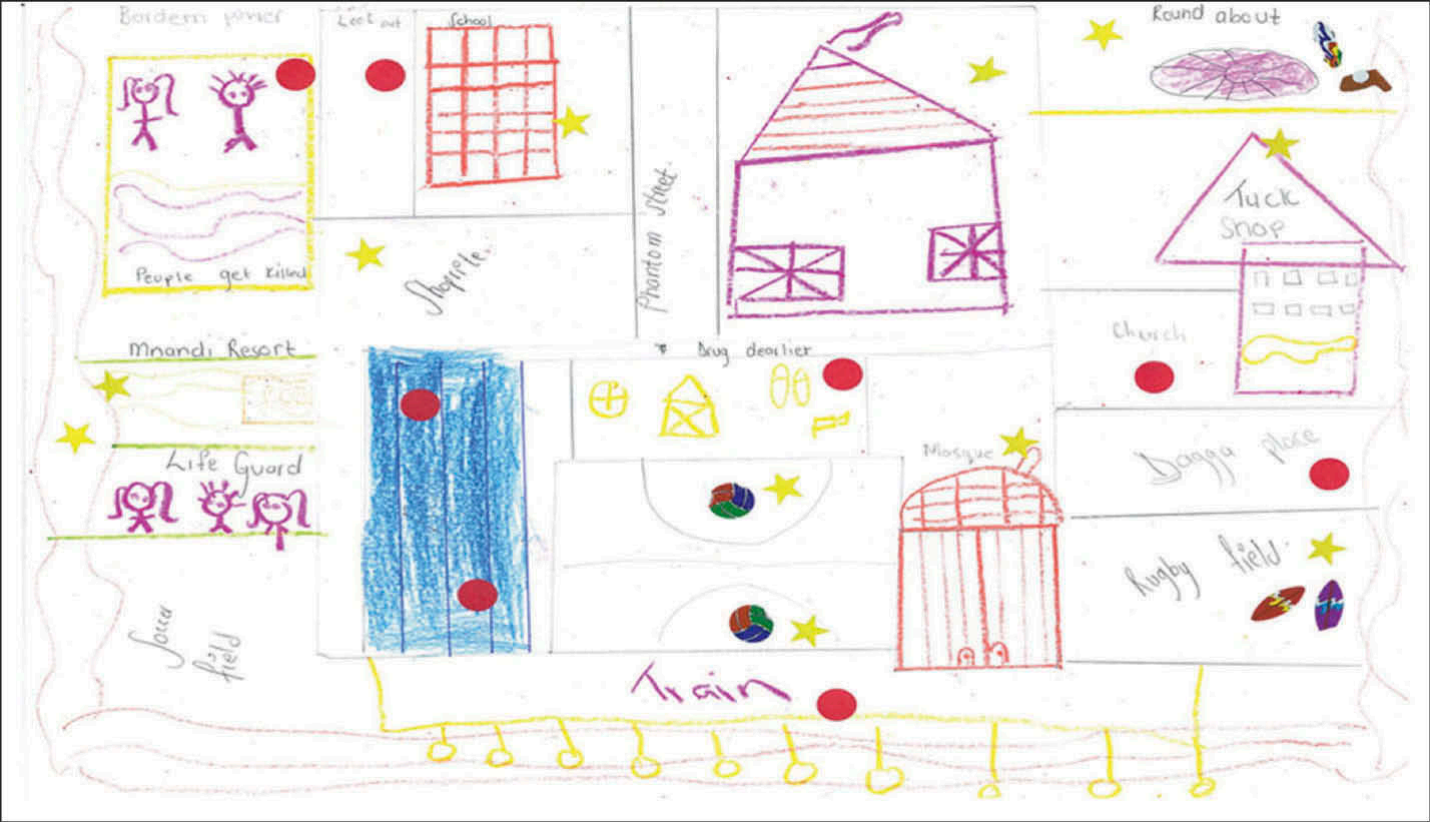
Map 4 shows a predominance of unsafe spaces for this participant, including known drug houses, the beach, the field close to home, and outside the church. Similar to the other maps, the school, soccer field, and mosque were identified as safe.

Beaches were another common unsafe natural space that children identified. Two of the schools, namely Gordon’s Bay and Mitchell’s Plain, were located within walking distance of a pristine coastline; however, owing to the danger of this space, the children from Mitchell’s Plain did not have any photographs of the beach present in their photovoice mission. In addition to beaches being unsafe, even when children are accompanied it is still unsafe, as one participant notes that you have to “Be your own lifeguard”. The children’s maps had two types of stickers: red stickers signified unhappy and unsafe spaces in their community, while gold stars signified safe and favourite places. When perusing the children’s maps in Figures 5 and 6 we see that there are numerous unsafe spaces which children identified. Children’s narratives accompanying their photographs demonstrated that while they feel that they “have no freedom”, and that they are unhappy about the circumstances of their lives, they show a resilience to cope with the stressors and threats they are faced with. Their limited range of mobility was expressed as frustrating for children, “go to school safe, go home safe”; thus reflecting the essence of a strict regimen, with little opportunity for fun and enjoyment. While discussing her map showing many unsafe spaces in nature in her community, a female participant declared that her lack of ability to engage in nature “makes me sad … makes me want to cry … it’s not enough for me”. Children’s exploratory ranges are close to home, affecting their ability to connect with nature and receive the benefits of engaging therein. The role of media reports of violence in the news was another factor which children mentioned as affecting their mobility, “because the news says this place and this place is dangerous”, resulting in parents worrying and becoming more paranoid and protective of children’s movements. What the children alluded to was an allegorical checklist, with preset conditions to ensure their safety outside their home; a lack of doing so might have severe consequences for their safety and well-being.

Other natural spaces such as the parks close to home were also categorized as unsafe by children, as these are places where criminals, such as drug dealers and gangsters, gather. As one female participant expressed “the drug dealers sit on the swings with guns”. In reference to her photographs, a female participant remarked that the “park there by us is very dangerous”, it is where “people are high”; she further notes that “Like Mandela said … it’s a long walk to freedom, to nature”. This statement was quite powerful as the participant used a revered political icon to bring across her feelings of being vexed by the inability to engage in nature due to the troubling and precarious nature of her community. Walking home from school, or walking to the tuckshop close to home, was particularly unsafe for girls, as they discussed incidents of being harassed and taunted by older boys and gangsters.

The critical concern of pollution was also evident in the children’s photographs, with a substantial amount of discussion focusing on how unsafe natural spaces are often littered with waste, rubbish, broken glass, and people burning tyres and other pollutants. Unsafe spaces, with nature perceived as the dangerous ‘other’, were often conflated with degraded, polluted spaces. Children’s photographs showed littered fields and beaches, making these already inaccessible childhood spaces more unreachable to them. Some children spoke about how they have to clean litter from their school grounds as they are seniors. Furthermore, they asserted that adults in their community advocate keeping their community clean but they do the opposite: “They always say you must lead by example, but they don’t do it.” Another participant added that the pollution “doesn’t bother the others because they not outside; it bothers us kids because we’re outside”.

Two extremities became manifest from children’s perceptions in reference to adults’ intrinsic need to safeguard children; the first was children’s acknowledgement that “They [adults] try to protect you”, to the second rejoinder that “It’s over-protective”. More broadly, these discordant views feed into issues of children’s social participation, circumvented mobilities, and the ability to explore their environments. Resonating with findings by Sancar and Severcan (2010), the findings in this study showed photographs of an absence of children in children’s places: with empty parks, empty sport fields, and degraded natural spaces in children’s communities, forcing them to remain in the confines of their home. These places were considered as ‘children’s places’ as these were the natural places which children sought out, but were not always able to make use of.

### Children’s favourite places in nature

**Figure 7.**
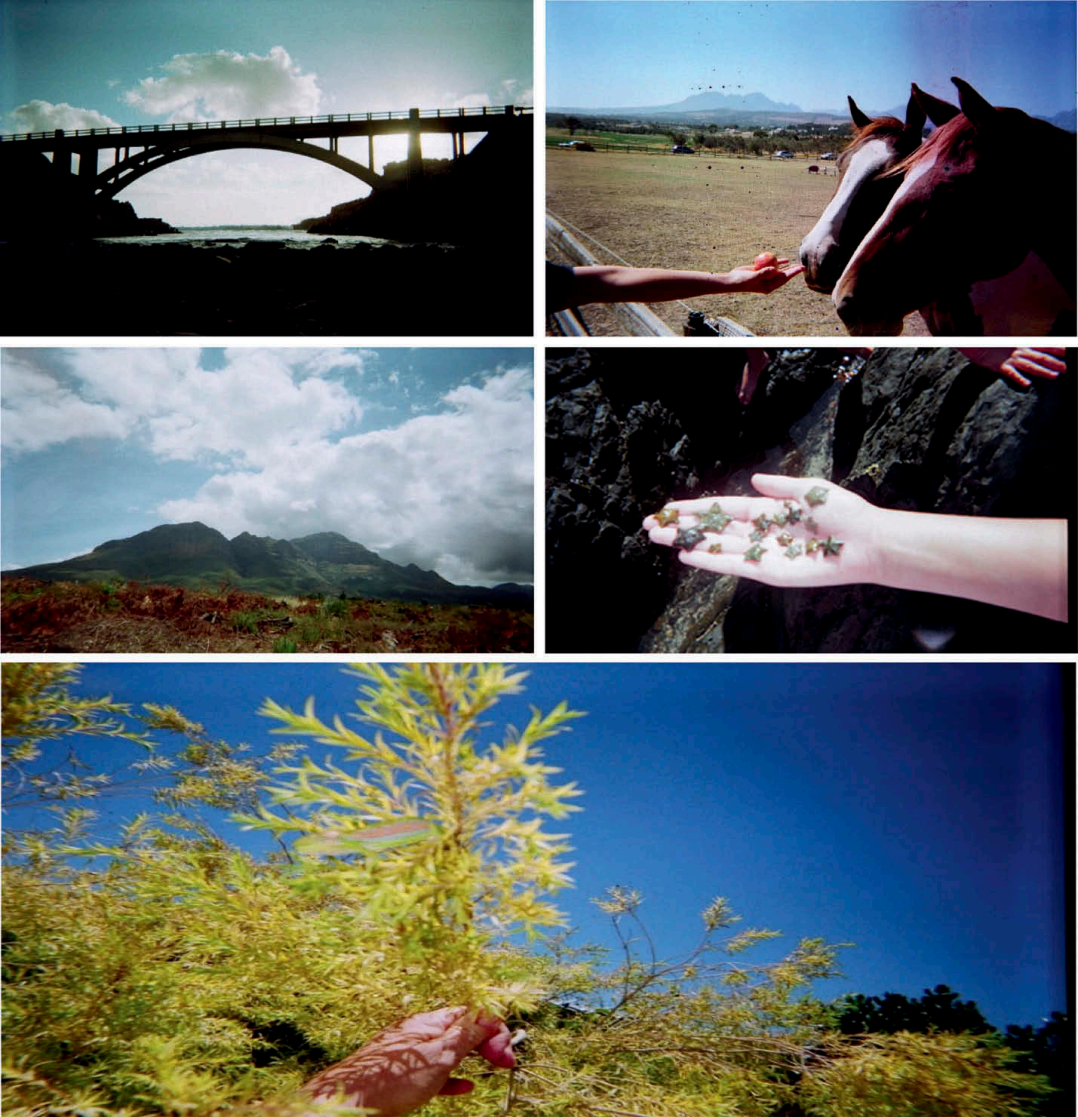
From the photographs, we can see that a picturesque river with a bridge, and being close to fauna (starfish, feeding horses, and chameleon, bottom) are among children’s favourite places in nature.

Children’s favourite places were more than just safe spaces in which they enjoyed engaging, as Abbott-Chapman and Robertson (2009, p. 419–420) aptly note, “favourite places are idealised constructs of places enjoyed and remembered which assist in regulating negative feelings and coping with perceived stress, whose emotional benefits are enjoyed irrespective of the frequency of visits”. The last point which these authors make was crucial for this study; that is, the affective component and children’s attachment to special places. In particular, that children’s special places can be revered without an abundance of experiences therein. For most children in the current study, discussions about their favourite places elicited a number of accompanying positive emotions; it made them feel happy, free, calm, and like themselves, and provided them the opportunity to spend time by themselves, or enjoy social connections with friends (Figure 6). Children’s favourite places were not always places they frequented. Many children mentioned cherished natural spaces which they visited on a family or school outing which made a lasting impression on them. Nature as children’s favourite place was evident in their photographs and maps, with many interesting findings emerging from the children’s discussions presented in Figure 7.

SES played a significant role in children’s identification with and engagement in natural spaces, with children from the middle SES community sharing several of their favourite places, while children from the low SES communities indicated far fewer. Children from Mitchell’s Plain and Stellenbosch often described ideal natural spaces, or natural spaces that were not always safe, or that they visited infrequently and were far from home. They mentioned that their favourite places were the parks, sports fields, an open field, gardens at home and school, the dam, and a burial park. Some children mentioned that they were happiest at school and at home, as these were the only opportunities they had to engage in some form of nature. School was cited by other children as a favourite place as they enjoyed sitting under the trees with their friends during recess. As most low-income schools in Cape Town have small grounds and are under-resourced, with many parents unable to pay a low rate of school fees, the play spaces for children in these schools are often asphalt play surfaces with a traditional playground. While traditional playgrounds are amenable to younger children’s play, for adolescents this is not appropriate, which emphasised in discussions with children. Adolescents, and younger children, need natural spaces at school where they can explore and learn about nature using ‘loose parts’ (Nicholson, 1971, as cited in Chawla, 2009). Children from Gordon’s Bay were not faced with the same restrictions as children from the two other locations. Children at this school had big school grounds, and a large and well-kept sports field to which they had access before and after school, as well as during their recess. There were apparent divergences in boys’ and girls’ favourite places: boys enjoyed surfing and playing on the sports field, but most preferred staying indoors and playing console or computer games; while girls preferred being outside, however, due to their risky neighbourhood were not always able to do so. Recollecting one of her favourite places, a female participant mentioned that “What makes me happiest is walking to the Steenbras dam and talking to my friend”.

Children’s environmental learning in these characteristically unsafe communities was stunted. This, however, did not mean that children did not appreciate or have an affinity toward nature. A favourite activity in nature that was prominent in all of the children’s discussions was participating in school camps. The impact of these camps on children’s environmental worldviews, and the resulting impact on their well-being, was well-established from children’s experiences. A female participant providing her recollection of the camp asserted that, “Since we come from the camp, before the camp I wasn’t interested in nature,” whereas after the camp the participant was extremely intrigued by nature and what it offers, “It feels like it’s interesting”, and more so how it made her feel, captured in her expression, “You want to relive that moment, over and over”. This participant highlights the manner in which she cherished a singular, capricious experience in nature. Children’s experience of a camp may have had a lasting effect on them as they were in a safe natural space, under the supervision of adults, and were able to learn about nature directly and explore this otherwise unfamiliar space. Further discussions about how nature made them feel led a participant to declare that her experience in nature made her feel that “Nothing was in your way”. This sense of freedom within nature was evident in many children’s photographs of open green space and natural scenery in their communities. The essence of “nothing” being in children’s way had both literal and figurative undertones. In a literal sense, large open space provided children with the opportunity to explore and roam freely, and figuratively, it was a safe natural space with no strangers or criminals impeding children’s mobility.

Children from Gordon’s Bay had much more varied favourite natural spaces. The beach was often deemed a favourite place, especially in summer when children could surf and bodyboard in the waves, play with starfish, or spend time with their family or friends. The photographs from these children were not merely of open fields or gardens as children discussed spending quite a bit of time exploring nature to find the perfect picture to capture their experience in it. Some of the photographs showed different types of reptiles and insects, which the photographers were very proud of taking. One participant also mentioned how she sometimes enjoys chasing birds on the field close to her home, one of her favourite places, which she also photographed. A male participant mentioned that one of his favourite places was his friend’s house, which is close to a farm. One of his photographs was of him feeding the horses apples on a visit to his friend. When asked whether he was afraid of the horse, he mentioned that he was not, and that he enjoyed spending time with animals and was not afraid of ‘wild nature’. Children also mentioned that they enjoy hiking in the mountains, which is good exercise as well, but is sometimes dangerous due to the presence of baboons. Hiking also allowed them to uncover and explore a cave in the area, among their favourite places. Those children who had the opportunity to engage in natural spaces and the ability to explore nature in their community were evidently environmentally knowledgeable and conscious. They spoke about various species of birds, dogs, and sea animals, with many advocating environmentally protective behaviours. The presence of pollution such as litter on beaches and in neighbourhood parks was flagged as a deep concern from children’s perspectives as it threatens the Earth, as well as humans, and sea creatures if the litter blows into the water.

It is evident from Extract 3 (below) that nature was a special place for children. Based on children’s discussions of their experiences in nature, it emerged that children from the middle SES community were more acquainted with nature, founded on more affordances to engage in safe natural spaces. A female participant stated that “I love exploring in nature …”, with many indicating that they enjoy spending time in nature. Conversely, for the children living in the low SES communities, nature experiences were infrequent. It was most often the literal backyard or garden nature wherein children’s experiences were constructed. Children’s understandings of nature were intertwined with the varied constructions of safety from the low and middle SES communities. These constructions of ‘superficial nature’ may be linked to the lack of access and experiences in nature of the children in these impoverished communities. Natural space was often made sense of as an ideal place of childhood (Malone, 2016). A participant indicated that nature encompasses components that grow outdoors; thus, “it wasn’t man made.” Children from one of the low SES communities revealed that safe natural spaces were less accessible, but included built places with ‘superficial nature’, which comprised the aquarium, a theme park, and a games centre. Notwithstanding the limited access and engagement in natural spaces, the key experiences that children derived from nature were vivid memories with positive meanings, exemplifying Winnicott’s (1960) concept of the ‘good enough hold’ (Adams & Savahl, 2016b). “Positive emotions experienced in natural spaces are therefore fostered, internalised, and espoused.” (Adams & Savahl, 2016b, p. 20). Further discussion about their favourite times spent in nature presented reiterated narratives of nature experiences, revealing the poignant but often shallow constructions and experiences in nature.

#### Extract 3

**Interviewer:** Do you go to any places where there is nature?

**Male participant:** Yes, yes.

**Male participant:** By the … aquarium.

**Male participant:** And … there where the water comes from the mountain.

**Male participant:** Waterfalls.

**Interviewer:** What are your favourite places in nature?

**Male participant:** Ratanga [An amusement park]. [All talk at once]

**Male participant:** Mine is Grand West[an entertainment centre and ice-rink] where there is lots of games to play.

**Male participant:** I like ice skating. Aquarium.

**Male participant:** You think Grand West is nature?

**Male participant:** For me it is.

**Male participant:** The ice is maybe nature.

(**Group 1: Session 2; Mitchell’s Plain)**

**Female participant:** Of everything that’s in the outdoors like the stuff that grew by itself it didn’t—it wasn’t man made.

**Female participant:** I think of it because I love exploring in the nature and like taking pictures of things that I don’t really know much about …

**Female participant:** Surfing.

**Female participant:** I just like laying on the grass and watch the clouds and the birds and the trees or something.

**Co-facilitator:** … how does that make you feel?

**Female participant:** Relaxed.

(**Group 2: Session 2; Gordon’s Bay)**

In a similar study exploring children’s discursive constructions of nature in Cape Town, Adams and Savahl (2016b, p. 11) note that:

… children from the differing SES communities evidently produced distinctive conceptualisations of nature which appeared to be influenced by the context and social milieu of their neighbourhoods, as well as their level of affordance to engage in nature. For the children from the low SES communities nature encompassed any space which possessed elements of nature … The conceptualisations of nature for the children from the middle SES community were markedly different—these children made sense of nature as a familiar space, and pointed more to nature being synonymous with ‘wild nature’ such as the forest, the mountain, and the beach, which were all places children frequented and displayed an intrinsic care for.

The children were evidently aware of issues around climate change, and discussed how all children and schools should become involved in environmentally friendly behaviours in their community, with the help of its residents. A female participant discussed how, in her family, “We’re quite environmental freaky!”, which was manifest in her map and photographs, which paid attention to natural detail in her favourite places as well as the environmental knowledge that she had. While she revealed that some of this knowledge was acquired in a few school subjects, her interest and intrinsic care for nature were predominantly gained and assimilated from her mother, as she stated that “My mom she really likes the environment and plants”. More so, in discussing a photograph she referred to “the solar-powered fairy lights” that her mother uses in the garden. This reference to the various ways of incorporating environmental concern speaks to a discursive theme of the influence of the intergenerational transmission of environmental consciousness of parents’ and other significant close family members, on children’s meaning-making (Chawla, 2006). This theme was also present in discussions with children from the other communities. Children spoke about how the garden at home was one of their favourite places, as they liked to plant things and maintain them. A male participant spoke about how one of his favourite times spent in nature was when he visited his grandparents and they spent all day gardening and learning about flora.

## Discussion

Sancar and Severcan (2010) note that, notwithstanding socio-cultural and contextual nuances in children’s spaces, their favourite places are inextricably linked to the memories and feelings these elicit from them. In this study, three themes emerged from children’s discussions about their special places in nature, namely: safe spaces in nature, unsafe spaces in nature, and children’s favourite places in nature. The SES of the communities in which children live was the determining factor shaping their childhood. For the children from the low SES communities, the places they most frequently inhabited were the safe places and spaces that they photographed and included in their maps, while the children from the middle SES community frequented their favourite places more often. The crucial difference between children’s lives in these two contexts was the pertinent issue of safety. Children from the two low SES communities, characterized by high levels of crime and violence (among the highest in the region), revealed feeling uneasy and unsafe when they were outdoors in their neighbourhoods.

The significance of context was apparent in children’s sense-making and discussions about their favourite places. Children’s understandings indicated that most areas in their community are unsafe, exemplifying the high levels of crime and violence in low SES communities in this context. Children’s neighbourhoods, the ‘dominant locality’ of their daily lives, were shown to be imbued with numerous concerns around their personal safety; these concerns were heightened for children from the low SES communities as they faced pervasive threats. Although the low SES neighbourhoods in this study are deemed among the most dangerous in the country, other local studies conducted in various contexts have identified safety as a ubiquitous concern for children across South Africa (Adams & Savahl, 2015; Isaacs & Savahl, 2013; Parkes, 2007; Savahl, Malcolm, et al., 2015; Swart-Kruger & Chawla, 2002). This evinces the nuances in children’s understandings of natural spaces owing to their distinctive neighbourhood geographies. A key consideration for this study resonates with McKendrick’s (2014) contention that “where children live interfaces with other factors to shape children’s well-being.” (p. 279). While he contends that where children live does not determine their well-being, it is crucial to note that in the context of South Africa, children’s residential geographies plays a key role in how they make sense of their lives. Addressing intersectionality is a key consideration here, as it was evinced that children’s constructions of nature were not only influenced by the “limited single axis notion” of gender, but also included social inequality and SES aspects of identity (Alanen, 2016, p. 158). This essentially points to the “intersectional structure of children’s lives” which influences how children made sense of their “lifeworlds” (Alanen, 2016, p. 159). Similarly, contrasting and divergent understandings of nature were presented by children, along with the related impact that engagement in nature has on their well-being. Children often constructed nature through binaries, with nature as a familiar and estranged place, a threatening and threatened space, or the dangerous ‘other’ and special place. These meanings attached to nature, however, were not independently assigned but instead displayed the intersection of varied understandings of nature owing to distinct personal experiences therein.

The divergent characteristics and social context of children’s environments can be considered in terms of Thomson and Philo’s (2004) notion of ‘classed spaces’. The idea of ‘classed spaces’ in a sense aptly captures the limits on children’s mobility in their neighbourhoods, how this has numerous impacts on their daily lives, and how they make sense of their experiences in private and public spaces. The term ‘class’ is employed to indicate the diverse social status, income, resources, and quality of life that children experience as a result of the area in which they live (Thomson & Philo, 2004). It further delineates the distinct constructions of nature and SWB that children from the two SES communities presented. An additional explication can be found in the concept of a ‘satisfaction paradox’ (Zapf, 1984, as cited in Olsen and Schober 1993), denoting people being satisfied irrespective of objectively unsatisfactory living conditions (Neff, 2007; Savahl, Adams, Isaacs, Hendricks, & Noordien, 2015). Children’s communities being constructed as ‘classed spaces’ brings with it several implications for children in terms of mobility, and access to safe public and natural spaces; this was further confounded by the lens of safety through which children negotiated their lives.

Despite their perilous environments, the children in this study displayed a resilience to cope with unsafe and risky spaces. What was striking in children's photographs, and particularly in their maps, was their motivations for walking a particular route to school, or to a friend’s house, which enabled them to circumvent a ‘dangerous place’. Thus, children coped with risk by evading unsafe and dangerous spaces. This resonates with Leonard’s (2007) contention that “Risks were avoided, confronted, negotiated and renegotiated” (p. 443). Children’s sense of worth appeared to be augmented when they reached a destination safely and were able to navigate their way through their neighbourhoods. Many of the children spoke about first-hand experiences of being bullied or harassed by gangsters in natural spaces, accompanied by feelings of anxiety. Garbarino, Dubrow, Kostelny, and Pardo (1992) note that in these perilous communities, danger, threat, and a sense of fear become the norm. Nonetheless, children were still able to enjoy leisure time with friends, at parks or sports fields which were monitored by adults. The gender differences that emerged from this study were contrary to a number of international studies, indicating that girls preferred to be outdoors and boys preferred to be indoors. Children from these communities were reliant on adults to ensure their safety, and were unable to explore nearby natural spaces such as beaches, dams, rivers, parks, and nature reserves. These safe and unsafe places portrayed in children’s maps and photographs serve as a representation of the ‘everyday worlds of childhood’ (Leonard, 2007).

Children’s narratives about their favourite natural places gave them an opportunity to reminisce and re-experience the feelings from that place. These special places provided children with a place of solace. Children from Gordon’s Bay, having access to safe natural spaces in their community, displayed an environmental knowledge akin to being familiar with nature, which was not apparent among children from the other communities. This knowledge was also evident in the children’s photographs, which captured ‘hidden’ nature and special natural spaces. In contrast, children’s photographs from the low SES communities depicted nature which they did not frequent, such as the park and the field, which Sancar and Severcan (2010) refer to as ‘spectator spaces’; while others took pictures of ‘superficial nature’ such as a plant nursery. This alludes to another key finding in terms of the varying ways in which the participants made sense of ‘nature’, and evinces the various constructions and contestations around the concept of nature as delineated in the literature (Macnaghtan, 1993; Linzmayer & Halpenny, 2013; Taylor, 2011). This relates to the assertion by Linzmayer and Halpenny (2013, p. 1) that experiences in nature involve intricate processes which amalgamate the “relational meanings we attach to those experiences”. This resonates with Macnaghtan and Urry’s (1995, p. 95) conjecture that: “there is no single ‘nature’, only natures. And these natures are not inherent in the physical world but discursively constructed through economic, political and cultural processes.” This highlights the notion that children’s relationship with nature is grounded in their subjective experiences in nature as well as affected by broader social and cultural factors (Linzmayer & Halpenny, 2014).

The findings further point to the dichotomy between ‘urbanized nature’ and ‘wild nature’. Many of the participants from the low SES communities, who had fewer experiences in natural spaces, made sense of all outdoor spaces as ‘nature’. Findings from a study by  Adams and Savahl (2016b) in South Africa evinced that children from differing SES communities and geographical locations had differing conceptions of nature, which were to a large extent influenced by access to safe natural spaces. Additionally, for some children natural spaces included things that grew outdoors and were not man made, while for children from one of the low SES communities, there was no distinction; instead, all built places with superficial aspects of nature came to encompass nature (Adams & Savahl, 2016b). Similarly, in the current study children’s understandings of and engagement in natural spaces were closely related to the SES of their community and concerns around safety, as well as broader social, political, cultural, and economic factors which affect children’s accessibility to and behaviour in nature, public spaces, and their special places (Skar, Wold, Gunderson, & O’Brien, 2016). Appended to the distinctions in terms of what nature meant to children was the overarching aspect of children’s use of differentiated spaces and places (Kjørholt, 2003). Children’s environments and spaces are not static but variable, as they are “but a set of dynamic factors, producing different outcomes for different groups of children.” (Ben-Arieh & Frønes, 2011, p. 250). This consideration is particularly significant in relation to the social inequality in South Africa, and how these differing conditions impact children’s realities and how they make sense of their lives, but also speaks to children’s ‘sense of place’ or place attachment. Scannell and Gifford (2010) put forward the Tripartite Organizing Framework encompassing three key dimensions of place attachment, namely person, psychological (process), and place dimensions. In relation to the current study, the dimension of the ‘person’ emphasizes that place attachment is greater when particular locales arouse personal memories (which is believed to further influence a stable sense of self) (Twigger-Ross & Uzzell, 1996); such as when places are linked to specific memories of experience. Therefore, akin to findings by Wals (1994), children in this study who showed greater familiarity with nature as a space were those who were able to engage in safe natural spaces and were from a more affluent community. Owing to their experiences, these children were able to differentiate between space and place. The children’s narratives from the middle SES community poignantly underscored Relph’s (1976) and Tuan’s (1977) notion of a space as something abstract, which advances into a place, a site of significant meaning for the child through lived experiences and the assignation of specific meanings to these. This provides support for individual experiences as a source of place attachment in addition to the physical characteristics of the place (Scannell & Gifford, 2010). This aspect of familiarity is encompassed in the concept of ‘place identity’ (Proshansky & others, as cited in Scannell & Gifford, 2010), the cognitive (psychological) component of place attachment. When a child identifies with a place, it entails a component of sense of self, whereby the child incorporates the place, and in this instance nature, within the self, and essentially fosters an ‘environmental identity’ (place component) (Clayton, 2003).

With South Africa being a signatory to the UNCRC, it is obligatory upon the government to take a more pronounced stance and action in bettering children’s lives. While the UNCRC speaks to a safe social environment for children (Articles 19 and 27), it does not directly mention the natural environment. The only article that speaks to the natural environment is Article 29, which states that a key goal of children’s education should be to enable them to protect the environment. Given the numerous benefits of children engaging in natural spaces for their psychological well-being and overall quality of life (Huynh, Craig, Janssen, & Pickett, 2013), Myers (2014) critically notes and advocates that access to nature should be regarded as a fundamental right of children. As children’s SWB has been shown to directly associate with their engagement and experiences in nature, it is crucial to increase children’s access to safe natural spaces in their neighbourhoods (Kerret, Ronen, & Orkibi, 2014).

## Conclusion and recommendations

This study explored visual representations of children’s lives, providing a different lens through which to understand children’s sense-making and attachment to favourite places. With natural spaces specified as children’s favourite places in this study, and the manifest advantages of children’s engagement therein, it becomes crucial to harness children’s access to natural spaces in their communities. In the current study, SES was a determining factor in terms of how children made sense of natural spaces (see also Adams & Savahl, 2016b; Sancar & Severcan, 2010). Safety emerged as a key aspect through which children made sense of their lives. High levels of crime and violence in children’s communities, and the accompanying concerns about safety, have limited children’s mobility and ability to explore natural spaces. As Sancar and Severcan (2010, p. 318) advance:

An accessible, child-friendly public realm plays a vital role in children’s socialization and development into responsible members of civil society, especially during periods of intense transformation, when stable social structures are shattered by the intrusion of new populations and lifestyles.

Although significant progress has been made in terms of legislation advocating and protecting children’s rights in South Africa, this has not culminated in safer environments for children which ensure personal safety in their daily lives. In developing contexts such as South Africa, participatory child-led initiatives such as the local Growing Up in Cities (GUIC) project, which was implemented in Johannesburg, to enhance children’s expertise and co-collaborators in key aspects about their life is crucial (Swart-Kruger & Chawla, 2002). Working towards building environmentally and child-friendly communities for children, with children as key contributors in the planning process using a child participation framework is decisively needed in South Africa. However, in the past decade, research on enhancing child friendly cities in the country has been absent. Nature should thus be a part of children’s everyday life so that they can reap its benefits, and in turn acquire environmental knowledge by becoming environmentally conscious and developing an environmental ethic which encourages sustainable development. Future research in the South African context should encompass participatory research with children from diverse contexts and regions, with children as consultants using participatory methods such as photovoice, community mapping, and walking interviews. Endeavouring to promote children’s well-being, a useful methodology was proposed by Benninger and Savahl (2016) with child experts using a ‘children’s delphi’. As the participants in the current study emphasized the importance of considering nature in the creation of a child friendly city, the intention of environmental awareness should be to enhance care for natural environments, on the basis of one’s own health and the well-being of others, as well as nature itself.
